# Structure of the bacteriophage PhiKZ non-virion RNA polymerase bound to a p119L open promoter analogue

**DOI:** 10.1107/S2052252525009273

**Published:** 2026-01-01

**Authors:** Chao-Sheng Chen, Natàlia de Martín Garrido, Maria Yakunina, Christopher H. S. Aylett

**Affiliations:** a Section for Structural and Synthetic Biology, Department of Infectious Disease Imperial College London London United Kingdom; b Department of Clinical Microbiology and Immunology, Gray Faculty of Medical and Health Sciences Tel Aviv University Tel Aviv Israel; c Institute of Cancer Research, 237 Fulham Road, London, United Kingdom; Max Planck Institute of Molecular Physiology, Germany

**Keywords:** bacteriophages, jumbo-phages, β subunit, β′ subunit, cryo-EM, ΦKZ, PhiKZ, RNA polymerase, σ factor, single-particle analysis

## Abstract

Bacteriophage ΦKZ (PhiKZ) was the first identified member of a family of massive bacterial viruses. ΦKZ infects *Pseudomonas aeruginosa*, which kills tens of thousands every year, and it therefore has potential as a bacteriophage therapy. On infection, ΦKZ forms a ‘nucleus’ to protect its genome by excluding host immune systems. This barrier means that it has had to become independent of the host transcriptional apparatus; it cannot simply recruit the host RNA polymerase (RNAP) to its promoters as it is excluded from the viral DNA, and therefore it expresses and imports its own non-virion RNA polymerase (nvRNAP). The ΦKZ nvRNAP, and related jumbo-phage RNAPs including that from bacteriophage AR9, are particularly noteworthy. Unlike typical viral RNAPs which are formed as only a single subunit, it is a non-canonical multi-subunit RNAP directly related to those from eubacteria, and more distantly eukaryotes and archaea. It encompasses four proteins representing patchwork homologues of the eubacterial β/β′ subunits, and a fifth that appears to have evolved from a σ factor, but no homologues of the α or ω subunits required for formation of a catalytically active complex in eubacterial RNAPs. Its mechanism of promoter recognition is also highly divergent; transcription is initiated from a site marked only by a tiny four-base consensus sequence co-located with the start site. We have resolved the structure of the ΦKZ nvRNAP bound to an open analogue of its cognate promoter, p119L, revealing that while the σ-factor-like subunit GP68 is involved in bubble stabilization, the sequence-specific promoter consensus sequence is bound between the lobe of the β-subunit homologue GP123 and the enzymatic core of the complex. Our results shed light on the differences between mechanisms of promoter recognition in the ΦKZ nvRNAP and canonical eubacterial RNAPs, and on the uniquely specialized features of bacteriophage transcriptional apparatuses in general.

## Abstract 2

Bacteriophage ΦKZ is a massive bacterial virus with potential as a therapy for *Pseudomonas aeruginosa*, which forms a bacteriophage nucleus in infected cells to exclude the host immune system, and transcribes its genes with its own, non-canonical multi-subunit RNA polymerase. The structure of this polymerase in the process of binding an analogue of its cognate promoter is resolved, revealing an unanticipated method of promoter consensus recognition involving the β-like, rather than the σ-factor-like subunit.

## 1. Introduction

Bacteriophage ΦKZ was the first bacteriophage classified as ‘giant’ due to its exceptional size (head diameter: 120 nm, tail length: 180 nm), encapsulating a huge, densely packed DNA genome of 280 kbp (Mesyanzhinov *et al.*, 2002 ▸). It is the prototypical member of a family of bacterial viruses distantly related to the *Myoviridae* (Krylov *et al.*, 2021 ▸; Krylov *et al.*, 2007 ▸; Krylov & Zhazykov, 1978 ▸). Bacteriophage ΦKZ infects *Pseudomonas aeruginosa*, which is an intrinsically antibiotic-resistant opportunistic pathogen of special concern for multi-drug resistance, posing a significant challenge to clinical practice (Lister *et al.*, 2009 ▸; Poole, 2011 ▸). Therefore, bacteriophage ΦKZ has been considered as a potential candidate for bacteriophage therapy due to its ability to effectively combat *P. aeruginosa* infection (Hall *et al.*, 2012 ▸; Can *et al.*, 2018 ▸; Pires *et al.*, 2015 ▸). Bacteriophage ΦKZ has a highly divergent life cycle and its massive genome encodes many proteins that would usually be expected to be provided by the host (Prichard *et al.*, 2023 ▸). To protect its genome from host immune systems it forms a membrane vesicle in the early stages of infection (Antonova *et al.*, 2023 ▸; Antonova *et al.*, 2024 ▸; Mozumdar *et al.*, 2024 ▸; Armbruster *et al.*, 2025 ▸) and a proteinaceous ‘nucleus’ within the host cytoplasm during the later stages (Chaikeeratisak, Nguyen, Egan *et al.*, 2017 ▸; Danilova *et al.*, 2020 ▸; Mendoza *et al.*, 2020 ▸). To manage the intracellular traffic during ΦKZ infection a tubulin cytoskeleton based on bacteriophage proteins is also constructed inside the cell (Aylett *et al.*, 2013 ▸; Chaikeeratisak *et al.*, 2019 ▸; Chaikeeratisak, Nguyen, Egan *et al.*, 2017 ▸; Chaikeeratisak, Nguyen, Khanna *et al.*, 2017 ▸).

The lifecycle of ΦKZ in *P. aeruginosa* is unaffected by the host RNA polymerase (RNAP) inhibitor rifampicin, implying that ΦKZ is independent of the host transcriptional machinery. While most bacteriophage-encoded polymerases are single-subunit RNAPs (ssRNAPs), ΦKZ is one of very few bacteriophages that encodes two non-canonical multi-subunit RNAPs (msRNAPs) (Campbell *et al.*, 2001 ▸; Ceyssens *et al.*, 2014 ▸; Sokolova *et al.*, 2020 ▸): a virion RNAP (vRNAP) that is injected with the bacteriophage genome during infection to transcribe early genes, and a non-virion RNAP (nvRNAP) responsible for transcription of all further genes needed for the assembly of new bacteriophages and the lytic cycle (Clark *et al.*, 1974 ▸; Sokolova *et al.*, 2017 ▸; Yakunina *et al.*, 2015 ▸). In the case of bacteriophage ΦKZ this independent transcriptional strategy may be favoured due to the isolation of the bacteriophage genome from the host transcriptional machinery at all stages, initially by the membrane vesicle and latterly by the bacteriophage nucleus (Antonova *et al.*, 2023 ▸), whereas in other cases of related non-canonical msRNAPs, such as bacteriophage AR9, the genome contains uracil in place of thymine, and this base is instead necessary for transcription initiation (Sokolova *et al.*, 2017 ▸).

Canonical msRNAPs exhibit a conserved double-ψ β-barrel (DPBB) domain in two large, related subunits, β and β′, in bacteria (Forrest, 2019 ▸; Iyer *et al.*, 2003 ▸; Lane & Darst, 2010*a* ▸; Sokolova *et al.*, 2020 ▸), however the β/β′ heterodimer is catalytically inactive without a dimer of α subunits and an ω subunit (Heyduk *et al.*, 1996 ▸; Minakhin *et al.*, 2001 ▸; Zaychikov *et al.*, 1996 ▸; Zhang *et al.*, 1999 ▸). Although this core (α_2_ββ′ω) is active, exchangeable σ subunits are required to initiate transcription from cognate promoters (Lane & Darst, 2010*a* ▸; Lonetto *et al.*, 1992 ▸). ΦKZ encodes two sets of genes that are homologous to the two largest subunits of eubacterial msRNAPs, β and β′ (Ceyssens *et al.*, 2014 ▸), but does not possess homologues of the α or ω subunits required for the assembly of a catalytically active enzyme in eubacterial msRNAPs (Heyduk *et al.*, 1996 ▸; Minakhin *et al.*, 2001 ▸). The ΦKZ vRNAP is formed by at least five subunits (GP178, GP149, GP180, GP80 and GP176) (Thomas *et al.*, 2016 ▸), whereas the ΦKZ nvRNAP is formed by four subunits (GP55, GP71-73, GP74 and GP123) which are homologous to the two largest subunits of eubacterial msRNAPs (β and β′), and a fifth, GP68, with no detectable sequence homology to any other protein, which has until now been thought to be responsible for promoter recognition and transcription initiation (Yakunina *et al.*, 2015 ▸).

We previously determined the ΦKZ nvRNAP holoenzyme structure in the absence of oligonucleotides (de Martín Garrido, Orekhova *et al.*, 2021 ▸), and its transcribing complex bound to an RNA/DNA template covering the downstream half of the transcription bubble (de Martín Garrido *et al.*, 2024 ▸). As in the architecture of eubacterial msRNAPs, the ΦKZ nvRNAP has β/β′-like subunits retaining most of the conserved structural elements needed for the reaction cycle and stabilization of the transcription bubble. The fifth subunit, GP68, which is found at the same location as bacterial σ factors and the AR9 promoter specificity factor (Fraser *et al.*, 2022 ▸; de Martín Garrido *et al.*, 2024 ▸), conserves some of the structural elements of these factors, suggesting that GP68 has probably evolved from a common ancestor with bacterial σ factors. In the ΦKZ nvRNAP transcribing complex structure (de Martín Garrido *et al.*, 2024 ▸), the GP68 linker shares features with eubacterial σ factors, therefore implying the likely conservation of the mechanism of promoter release. GP68 contacts upstream DNA through an extended loop of its C-terminal region in a different manner to the situations in eubacterial counterparts, which stabilize their promoters’ -35-element through a helix-turn-helix motif, while AR9 utilizes a pseudo--35-element-binding motif for a similar purpose (Fraser *et al.*, 2022 ▸). The structure of the AR9 promoter complex revealed a related set of split β/β′-like subunits with similar features, noticeably the lack of a β′ rudder and β-flap-tip helix in comparison to eubacterial msRNAPs, while AR9 also lacks the β′ subunit Zn binding domain. These two nvRNAP complexes are relatively disparate, having an RMSD of 4.4 Å over the superimposable regions (88% of the sequence). AR9 also possesses a homologous σ-factor-like subunit, but exhibits a uracil-specific template-strand mechanism of promoter recognition (Fraser *et al.*, 2022 ▸). Such a mechanism is not applicable to bacteriophage ΦKZ, however, since this base is not present in its DNA. Bacteriophage AR9 and ΦKZ nvRNAPs therefore appear very likely to share common conserved core transcriptional mechanisms but must have divergent promoter recognition mechanisms (Supplementary Figure 1; Supplementary Figure 2).

Early RNA-sequencing analysis and *in vivo* primer extension experiments on ΦKZ promoters showed that the late promoter transcribed by ΦKZ nvRNAP was associated with very short, weakly defined motifs (Ceyssens *et al.*, 2014 ▸). Furthermore, the conserved late ΦKZ promoter motif required for nvRNAP transcription is located immediately upstream of the transcription start point, in stark contrast to the −10 promoter consensus element or the TATA box location in eubacterial or eukaryotic promoters, respectively (Lane & Darst, 2010*b* ▸; Paget & Helmann, 2003 ▸). ΦKZ nvRNAP appears to recognize a late promoter with an overly short consensus 5′-TATG-3′ that overlaps with the transcription start point at the 3′-terminal guanosine. It has been conclusively shown that this consensus sequence is essential, as substitutions in the motif abolish or massively reduce the efficiency of transcription (Yakunina *et al.*, 2015 ▸). The high specificity of nvRNAP to late bacteriophage ΦKZ promoters is therefore surprising, however, given the relatively high frequency with which 5′-TATG-3′ appears within the ΦKZ genome. Experiments in which large regions of sequence around the high-efficiency promoter p119L were swapped out to generate hybrid promoters have shown that the efficiency of transcription is significantly affected by sequences both upstream and more notably downstream of the start site, even though no consensus sequence or base-pair propensity is visible (Yakunina *et al.*, 2015 ▸). Taken together, these results suggested that ΦKZ nvRNAP has a novel promoter recognition mechanism and DNA melting strategy to fulfil its role in supporting ΦKZ bacteriophage propagation in comparison to canonical msRNAPs.

In this study we set out to determine the molecular mechanisms underlying promoter specificity in the ΦKZ nvRNAP transcription. To this end we have determined the 2.59 Å cryo-EM structure of the ΦKZ nvRNAP in complex with a p119L open promoter analogue generated via mismatches in the template strand but containing the intact −3 to +1 consensus motif on both strands. In this complex, GP68 is involved in destabilizing the B-form DNA helix at the DNA channel exit, implying a role for the σ-factor-like subunit in DNA melting and bubble formation. Surprisingly, however, the β-like subunit GP123, not GP68, directly binds the 4 bp promoter motif 5′-TATG-3′ of the non-template strand under the β-lobe, and therefore the β-like subunit is responsible for consensus sequence-specific promoter recognition during ΦKZ nvRNAP transcription. This observation further corroborates the complete loss of configurability within the ΦKZ nvRNAP implied by the embedding of the σ-factor-like subunit as an integral member of the complex, and supports the conclusion that bacteriophage RNAP evolution is driven towards complete promoter specificity. No further sequence-specific interactions are implied by the structure, suggesting that the propensity of stretches of DNA to undergo initial strand separation may well provide the remaining specificity for ΦKZ late promoter recognition.

## 2. Results and discussion

### 2.1. Iterative optimization generated a p119L open promoter analogue suitable for cryo-EM and a 2.59 Å structure was resolved from its complex with the ΦKZ nvRNAP

In our previous study, we resolved the structure of the ΦKZ nvRNAP transcribing a short RNA oligonucleotide from the p119L promoter (de Martín Garrido *et al.*, 2024 ▸). In this case the complex was stable, binding was confirmed by electrophoretic mobility shift assay, and the transcribing complex was sufficiently stable that it survived downstream processing. On resolution of the structure, the downstream DNA and active site DNA–RNA hybrid proved to be very well ordered, while upstream DNA was weakly visible. It has proven significantly more challenging to obtain an ordered promoter complex. DNA binding assays have generally been unhelpful for optimization as positive assay results are obtained for most oligonucleotides, but these do not necessarily translate into the visualization of ordered DNA, and promoter complexes do not necessarily survive the gel-filtration chromatography desirable for additional purification. Therefore, optimization of the p119L promoter analogue for binding was approached by cryo-EM reconstruction of successive complexes until well ordered DNA was recovered in screening structures. The cognate promoter sequence alone does not yield ordered ΦKZ nvRNAP–DNA complexes, which is not unexpected given that many eubacterial msRNAP open-promoter structures have required stabilization through the provision of targeted mismatches (Bae *et al.*, 2015 ▸; Zuo & Steitz, 2015 ▸). Given the template-strand uracil-based promoter recognition known to occur in the homologous AR9 nvRNAP (Fraser *et al.*, 2022 ▸), oligonucleotides bearing the template consensus only within an artificial bubble generated by mismatches were trialled; however, these also failed to yield structures with ordered DNA. Provision of the consensus sequence on both strands within a long double bubble generated by mismatches on either side of the consensus region yielded structures exhibiting weakly ordered DNA, and shortening of this bubble to reduce freedom of movement (finalized at five bases – oligonucleotides spanning −38 to +37 – Supplementary Figure 3) resulted in reconstructions exhibiting sufficiently well ordered DNA to proceed to high-resolution data collection. Samples were prepared on graphene-oxide films using similar parameters to those that had proven successful for the previous transcribing structure, and high-resolution data were collected using a 300 kV microscope (Supplementary Figure 4). Clean-up, selection for particles occupied by the p119L analogue DNA oligonucleotide and parameter optimization (Supplementary Figures 5 and 6) yielded a structure resolved to 2.50 Å by independent half-set reconstruction (FSC = 0.143). We sorted for the most conformationally stable structure and strongest density representing the p119L analogue DNA oligonucleotide, which entailed discarding particles in which the trigger-loop insertion domain had become ordered against the β-lobe as in the ΦKZ nvRNAP transcribing complex, and resolved a final structure, with better order within the peripheral domains and DNA of interest for promoter binding, to 2.59 Å by independent half-set reconstruction (FSC = 0.143) (Supplementary Figures 6 and 7).

### 2.2. The overall structure during promoter binding is similar to that of the transcribing ΦKZ nvRNAP complex, however the N-terminal domain of GP68 and upstream DNA are substantially better ordered while downstream DNA is less well ordered

**Figure 1:**
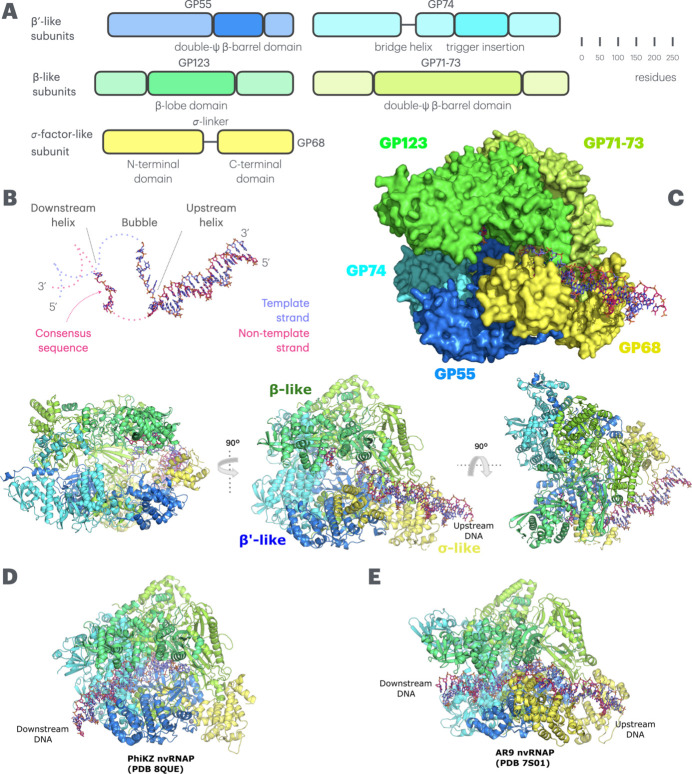
Overall structure of the ΦKZ nvRNAP bound to the ΦKZ p119L open promoter analogue. (A) Primary structure schematic for the ΦKZ nvRNAP protein complex. Key domains are highlighted and labelled. The β-like subunits, GP123 and GP71-73, are shown in lime green and chartreuse respectively, the β′-like subunits, GP55 and GP74, are shown in marine blue and cyan respectively, and the σ-like subunit, GP68, is shown in yellow. Template strand DNA is shown in purple, while non-template strand DNA is shown in magenta. This colour scheme is preserved for all figures showing the ΦKZ nvRNAP, while homologous subunits in the AR9 nvRNAP are shown according to an identical colour scheme to preserve orientation for the reader. (B) The paths of the template and non-template strands are shown without accompanying protein from the same perspective as in the panel below. Their low-resolution continuation and linkage are shown by dotted lines. Supplementary Figure 8 shows the density supporting this tracing, which is in agreement with our structure of the transcribing ΦKZ nvRNAP complex. (C) Top right panel: Overview of the ΦKZ nvRNAP–p119L open promoter analogue complex in surface representation. Left panel: side view of the complex in cartoon representation from the angle of the DNA entrance channel. Central panel: front view of the complex from the angle of the N-terminal domain of GP68. Right panel: Top view of the complex from the angle of the β-lobe of GP123. The rotations relating the central panel to the side panels are shown. (D) The transcribing ΦKZ nvRNAP complex is shown for comparison. Both this structure, and that in panel (E), are shown from the same perspective as the central panel of (C). (E) Comparison with the fork-DNA-bound crystal structure of the AR9 nvRNAP (see also Supplementary Figure 2).

**Figure 2:**
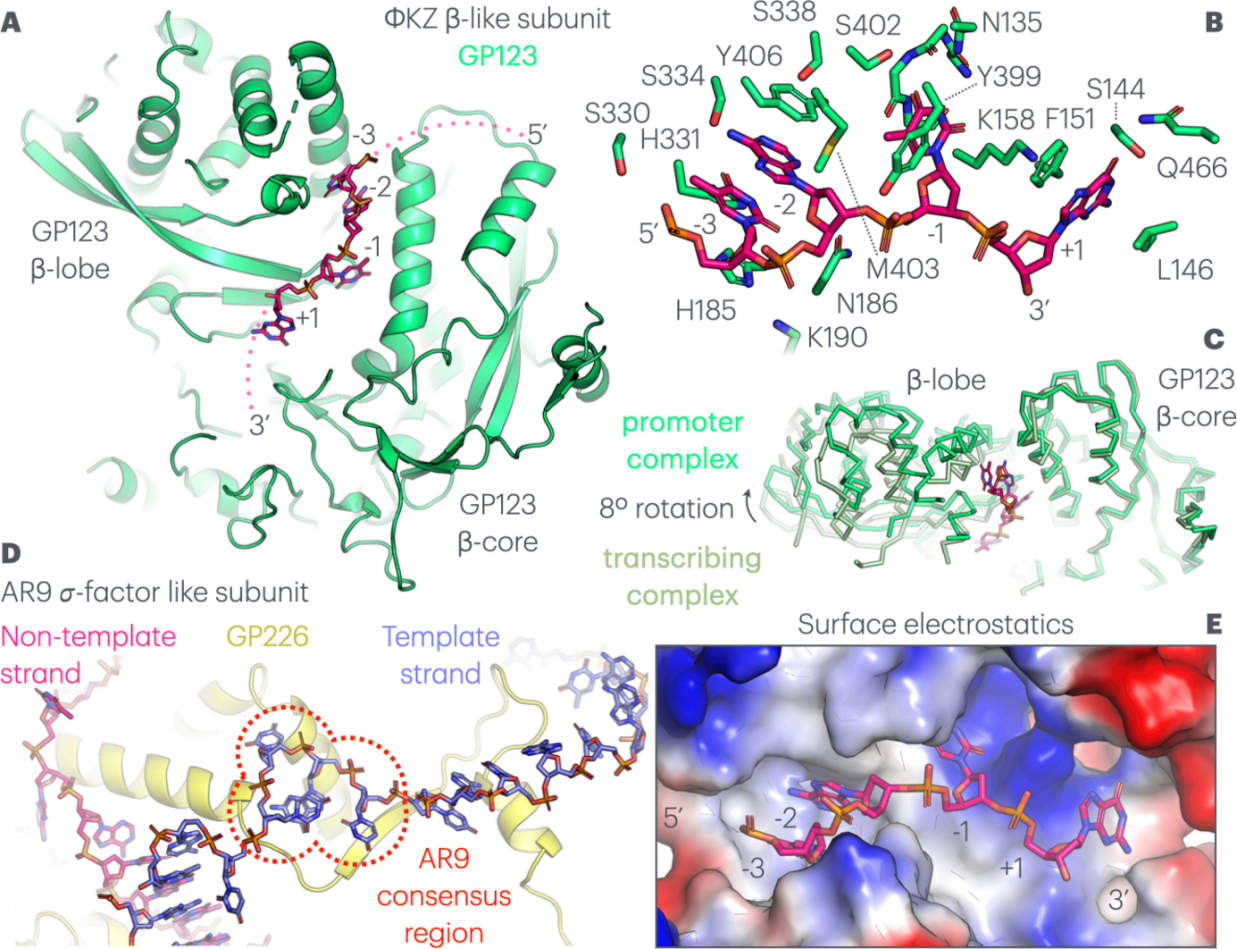
Sequence specific binding of the 4 bp promoter consensus region within the non-template strand by the β-like subunit GP123. (A) Internal view of the non-template strand 5′-TATG-3′ consensus sequence running through the surface pocket within the β-like subunit GP123, between the β-lobe and well ordered polymerase core. Low-resolution continuations of the DNA chain are shown as dotted lines, while the modelled bases are labelled. (B) The structure of the binding pocket around the non-template strand. Key interacting side chains and stretches of backbone from GP123 are shown and numbered, while the consensus nucleotides are labelled. (C) The rotation of the β-lobe against the GP123 core in the ΦKZ nvRNAP promoter analogue and transcribing structures (in darker green), showing the 8° rotation opening the pocket for consensus DNA sequence binding. (D) The uracil-dependent binding mode of the AR9 nvRNAP to its own four-nucleotide consensus sequence (highlighted within a dotted red enclosure) is shown for comparison. This takes place principally in a pocket within the σ-factor-like subunit, rather than the β-like subunit, and towards the template strand, rather than the non-template strand, and is highly divergent from ΦKZ promoter consensus recognition. (E) Electrostatic isosurface of the GP123 pocket, showing the deep intrusion of the bases and charge complementation of the backbone phosphate residues and base functional groups. Protein and DNA are visualized according to the Fig. 1 ▸ colour scheme.

When low-pass filtered to low resolution (10 Å) so that all features, even those at very low local resolution, are visible, the structure of the ΦKZ nvRNAP bound to a promoter analogue of p119L appears highly similar to that of the ΦKZ nvRNAP transcribing complex [Fig. 1 ▸(A)–(D); Supplementary Figure 8]. In particular, it is noteworthy that the general paths of the upstream and downstream B-form helical DNA outside of the DNA channel leading to the active site are conserved [Fig. 1 ▸(B)–(D)]. A more detailed consideration of the protein components at higher resolution reveals that the density is extremely well resolved in the core, with holes within aromatic-ring side chains visible in places (Supplementary Figure 9). The Cα RMSD with respect to the ΦKZ nvRNAP transcribing complex is 1.397 Å, which is indicative of non-trivial differences, but demonstrates that the majority of the modelled structure is very similar in each case. High-resolution consideration also reveals two major conformational changes in the protein elements of the complex. Firstly, the trigger-loop insertion domain is almost entirely disordered at higher resolution [Fig. 1 ▸(C)–(D); Supplementary Figure 6]. While this was the result of a trade-off made during sorting for better order of the p119L promoter analogue oligonucleotide and GP68 N-terminal domain (NTD), it is notable that the disordered state promotes GP68 stability. This appears to be because the β-lobe of GP123 is accommodated towards the trigger-loop insertion domain when it is ordered against it. Secondly, the β-lobe of β-like subunit GP123 is substantially displaced, rotating outwards and away from the remainder of GP123 within the well ordered core of the ΦKZ nvRNAP complex to accommodate DNA from the non-template strand [Fig. 2 ▸(A)/(C)]. We also observed significantly better order for the GP68 N-terminal domain in comparison to the previous ΦKZ nvRNAP transcribing structure, which is indicative of tighter binding and concomitant reduced rotational freedom [Fig. 1 ▸(C)]. Both these regions (of GP123 and GP68) could only be partially modelled due to low (>4 Å) local resolution at the peripheries; however, this is a significant improvement upon the situation in the ΦKZ nvRNAP transcribing complex structure, in which this region of GP68 could only be fitted with an unrefined *AlphaFold* model (Abramson *et al.*, 2024 ▸) [Fig. 1 ▸(D)]. Similarly, we observed significantly better order of the upstream B-form helical DNA, which is bound by the GP68 N-terminal domain, in comparison to the previous ΦKZ nvRNAP transcribing structure, in which it could not be modelled and only an idealized B-form helix fitted [Fig. 1 ▸(B)–(D)]. These two components move together, and their stabilization during promoter binding is notable as this is typically where promoter recognition occurs through σ factors in canonical eubacterial msRNAPs (Lane & Darst, 2010*b* ▸), even though this is not the case for ΦKZ. In contrast, however, the downstream B-form helical DNA and DNA within the active site region of the DNA channel are very weakly ordered compared with the previous ΦKZ nvRNAP transcribing structure, in which they were held more strongly in place by the binding of the DNA–RNA hybrid within the active site, which is not yet formed in the promoter complex; neither region can be modelled due to low local resolution [Fig. 1 ▸(B)].

### 2.3. While the DNA entrance and exit paths are conserved, the paths of the separated strands through the active site channel are quite different during promoter binding

**Figure 3:**
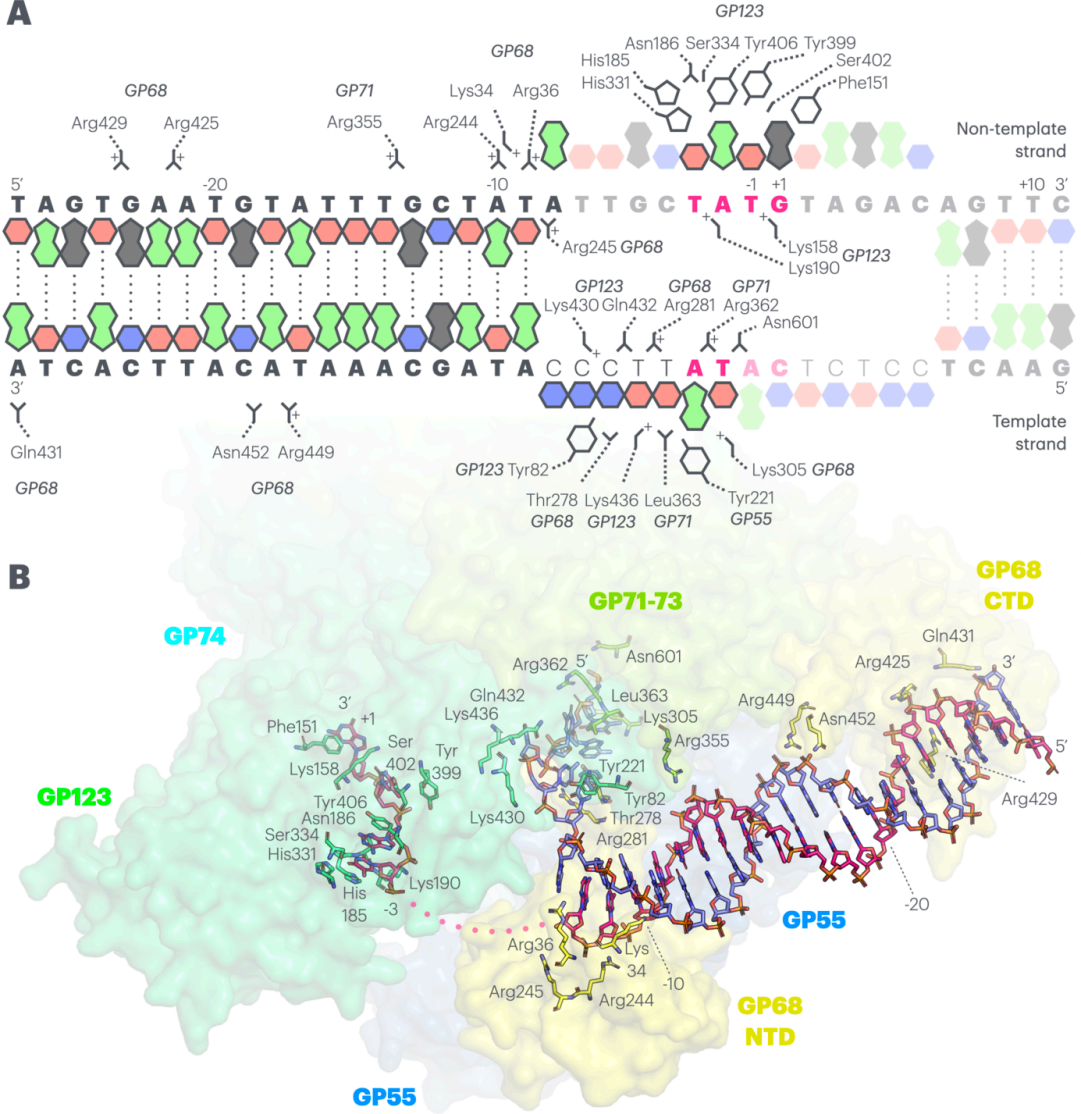
Schematic illustration of the binding elements from the ΦKZ nvRNAP interacting with the ΦKZ p119L open promoter analogue DNA. (A) The resolved region of the p119L open promoter analogue is shown as cartoon purine and pyrimidine bases in the standard DNA colour scheme (A: green, T: red, G: black, C: blue), with regions that are too weakly resolved to be modelled faded and lacking outlines. The p119L DNA sequence is shown in bold capitals, with the non-template strand above and template strand below, while the mismatches introduced to force bubble formation are shown in thin capitals. The 4 base-pair consensus sequence is shown in bold magenta text. Numbering is according to the transcription start site. The exposed bases in the region forming the bubble are shown with flipped out cartoons. Key interactions are shown through side chains connected to the relevant residue and protein name. Interactions with the phosphate backbone are shown reaching toward the appropriate letter in the sequence, while interactions with bases are shown contacting the relevant base cartoon. (B) DNA and contacting residues from panel (A) are shown within a transparent surface representation for ease of orientation. Stick representation is used with CPK colouring, and the labelling of individual residues is identical to that shown in panel (A).

**Figure 4:**
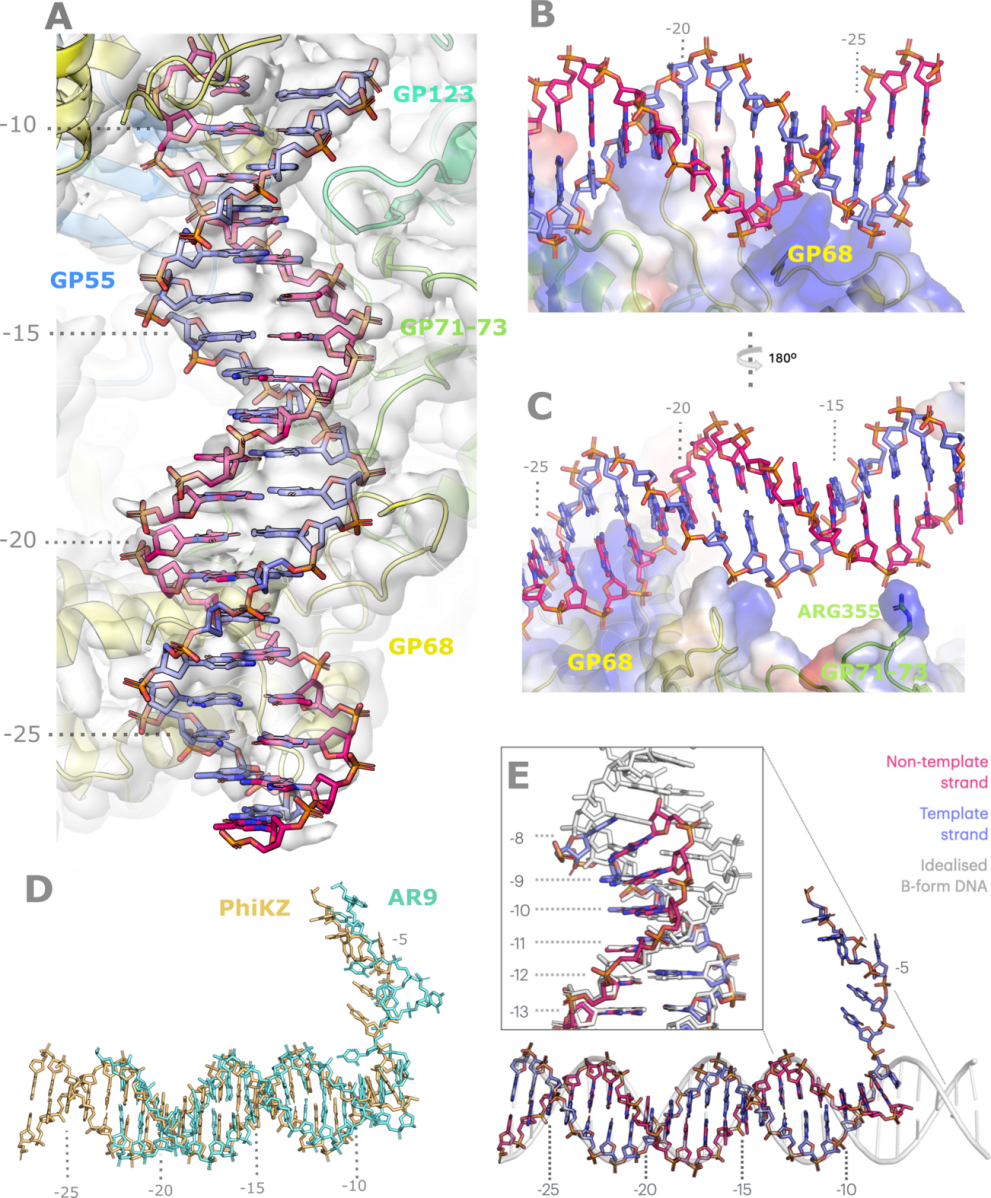
Stabilization and deformation of the upstream B-form DNA by the σ-factor-like subunit GP68. (A) The upstream double-helical DNA up to the formation of the bubble is shown in overview within a transparent isosurface of the experimental map. Base numbering has been superimposed for orientation. (B) Complementation of the phosphate backbone in the region −17 to −24 by predominantly positively charged residues from GP68. The electrostatic isosurface is rendered transparently to illustrate the charge complementation of the phosphate backbone. Blue indicates positive charge, red negative and white neutral, and this colour scheme is preserved for all other electrostatic isosurfaces rendered within this article. (C) Complementation of the phosphate backbone in the region −12 to −24 by predominantly positively charged residues from GP68 and GP71-73. The electrostatic isosurface is rendered transparently to illustrate the charge complementation of the phosphate backbone. Panels (B) and (C) are rotated by 180° from one another as indicated between them. (D) Comparison of the naked p119L promoter analogue DNA with the position of the upstream B-form DNA bound by GP226 in the AR9 structure, which is somewhat similar in situation and conformation. DNA bound to the ΦKZ nvRNAP is shown in gold, while that bound to the AR9 nvRNAP is shown in cyan. (E) Comparison of the deformed DNA bound by the N-terminal domain of GP68 to idealized B-form helical DNA (shown in white), detailing the helix approaching the promoter bubble. Inset is an expanded view of the region indicated with dotted lines, highlighting the deformation from −8 to −11. All protein and DNA is visualized according to the Fig. 1 ▸ colour scheme unless otherwise stipulated.

While only the upstream B-form helical DNA and parts of the independent strands within the DNA channel can be modelled directly [Fig. 1 ▸(B); Fig. 3], the full path of the non-template strand, and all except for a short region around the start site of the template strand, can be traced in filtered maps (Supplementary Figure 8). As previously mentioned, the paths of the B-form helical DNA upstream and downstream are very similar to the ΦKZ nvRNAP transcribing complex, and it is noteworthy that they also superimpose with those in the AR9 nvRNAP–fork DNA complex, even though the upstream DNA elements of this complex are non-cognate and feature a chain break at the juncture with GP226 [Fig. 4(D)]. Viewing the DNA from upstream to downstream, the point of bubble formation, where the B-form DNA helix breaks into separated strands, coincides with the point of contact with the GP68 N-terminal domain [Fig. 4(A)–(E)]. At this juncture the template strand DNA extends into the active site DNA channel, remaining surprisingly well ordered against the wall of the channel. The path taken begins broadly similarly to that in the ΦKZ nvRNAP transcribing complex but deviates towards GP55 and the GP68 linker at roughly the point at which it would join the DNA–RNA hybrid in the transcribing complex, before becoming too disordered to trace at roughly base +1 [Fig. 1 ▸(B); Fig. 4]. The AR9 nvRNAP–fork DNA complex undergoes promoter recognition in this region and thus forms a very different structure [Fig. 2 ▸(D); Fig. 4(D)]. It is important to note that while there are several stabilizing and stacking residues complementing this conformation, this strand is non-cognate due to the introduction of mismatches to force bubble formation, and therefore this accommodation is more likely to represent a general ordered pathway for exiting DNA (Fig. 3 ▸). There is no ordered template strand DNA visible at the active site, and the template strand can only be visualized once again behind the base of the bridge helix just before it reunifies with the non-template strand to form the downstream B-form DNA helix (Supplementary Figure 8). It is also notable that we observed similar disorder within the GP68 linker as in the ΦKZ nvRNAP transcribing complex, suggesting that this element is weakly ordered in general, rather than that it had become disordered as a result of transcription initiation. In comparison with the well ordered template DNA path entering the DNA channel, the non-template DNA strand becomes weakly ordered at the division into the bubble. It passes along the periphery of the core regions of GP123, in a conformation that must be extended to cover the relevant distance, before entering the cleft between the β-lobe and core where it becomes well ordered once again for the four bases of the consensus sequence, which could be modelled [Fig. 2 ▸(A)–(C); Supplementary Figure 8]. At this point the path of the backbone arcs downwards from GP123 towards GP55, meeting the template strand once again at the base of the bridge helix to reunite in a B-form DNA helix at the end of the bubble. The non-template strand therefore once again describes a notably different path from either the ΦKZ nvRNAP transcribing complex or the AR9 nvRNAP–fork DNA complex, but this route is in principle compatible with a reorganization of their non-template DNA.

### 2.4. GP68 stabilizes the backbone of the B-form DNA outside of the DNA exit channel, but deforms the B-form DNA immediately upstream of the site of strand separation

As previously noted, the upstream B-form helical DNA is found in approximately the same position in all three of our structures. We observe an almost perfect B-form DNA helix from base pair −11 up to the point at which the DNA begins to become disordered relative to the enzymatic core at roughly −30, and were able to model nucleotides up to base pair −27 [Fig. 3 ▸; Fig. 4 ▸(A)/(E)]. This DNA is ordered against the enzyme and stabilized by three interacting patches [Fig. 4 ▸(A)–(C)], in contrast to the situation in the AR9 nvRNAP–fork DNA complex, in which there is only a single stabilizing interaction between the phosphate backbone and a positively charged loop in the GP226 C-terminal domain (CTD). The first interaction made by the ΦKZ nvRNAP matches that made by AR9; a positively charged surface loop from the GP68 C-terminal domain, containing two arginines (Arg425 and Arg429), becomes partially ordered as it complements the negatively charged phosphate backbone around −22/−23 of the non-template strand [Fig. 4 ▸(B)]. The second stabilizing interaction is also contributed by the C-terminal domain of GP68, but in this case by the β-hairpin extending along the flank of GP71-73 (also referred to as the extended loop), which is tipped by an arginine and asparagine (Arg449 and Asn452) complementing the phosphate backbone at around −18/−19 of the template strand, closer to the bubble [Fig. 4 ▸(B)–(C)]. Finally, the third interaction with the B-form helix is contributed by GP71-73 rather than GP68, substantially closer to the site of strand separation, by Arg355, which is extremely well ordered and complements the phosphate between G −13 and T −14 on the non-template strand [Fig. 4 ▸(C)]. All three interactions are with the phosphate backbone, predominantly through positively charged residues, and this renders them unlikely to have any sequence specific character. The σ-factor-like subunit, GP68 in the ΦKZ nvRNAP and GP226 in the AR9 nvRNAP, makes the only well ordered contacts with the DNA as the template and non-template strands separate to form the bubble on the upstream side of the active site. As it approaches the point of bubble formation, the B-form DNA helix becomes substantially deformed in the ΦKZ nvRNAP promoter analogue structure [Fig. 4 ▸(E)]. This could not be resolved in the ΦKZ nvRNAP transcribing complex, as this region was of low local resolution, and is not conserved in the AR9 nvRNAP structure, which does not experience strong deformation, and is composed of two non-complementary forked-DNA molecules which cannot yield a connected bubble [Fig. 4 ▸(D)]. While the template strand remains approximately B-form and runs directly into the DNA channel on this trajectory, the backbone of the non-template strand runs through a positively charged groove on the surface of GP68, complemented by lysine and arginine residues (Lys34, Arg36, Arg244 and Arg245), which bends it backwards towards the DNA channel from −11 onwards until the first mismatch results in the stable bubble at −8 [Fig. 3 ▸; Fig. 4 ▸(A)]. This deformation certainly contributes towards changing the path of the non-template strand to match the required geometry of the DNA channel, and quite probably contributes to transcription bubble opening through GP68 more generally, by placing strain on the inter-strand base pairing at the point of separation.

### 2.5. GP123 sandwiches the key consensus sequence marking ΦKZ late promoters in a newly identified pocket between the β-lobe and the main body of the ΦKZ nvRNAP

Moving downstream, after strand separation, the non-template strand in the ΦKZ nvRNAP transcribing complex travels below the juncture of the β-lobe and the highly ordered enzymatic core towards the bridge helix. In the AR9 nvRNAP–fork DNA structure, it is ordered across the surface of GP226 and must follow a similar path below the β-lobe *in vivo* to reunite with the template strand. In the ΦKZ nvRNAP promoter complex, the consensus sequence between bases −3 and +1 is located here within the non-template strand, beneath the β-lobe of GP123 [Fig. 2 ▸(A)]. It is noteworthy that GP123 is particularly divergent from eubacterial β subunits, even more so than the other ΦKZ nvRNAP proteins, sharing only a few conserved motifs (Yakunina *et al.*, 2015 ▸). As previously covered, the β-lobe of GP123 is rotated upwards, away from the DNA channel, in relation to its position within the ΦKZ nvRNAP transcribing complex. The entire edifice is rotated by just under 8° relative to the remainder of GP123 as a rigid body, yielding ~4 Å deviation at the cleft in comparison with the transcribing complex structure [Fig. 2 ▸(C)]. This rotation opens up an elongated pocket, wholly within GP123 and incomplete in the transcribing complex, that accommodates the backbone and four bases of the consensus sequence [Fig. 2 ▸(C)/(E)]. Notably, the bases adopt a splayed conformation jutting into the pocket, consistent with sequence specificity, while the backbone is accommodated on the outside edge, and is less well ordered in the density, with the charges on the phosphates complemented by lysines (Lys158 and Lys190). Within the pocket, T −3 and A −2 are stacked together, while the other two bases are widely separated [Fig. 2 ▸(B)]. The pocket is highly complementary, with at least partial stacking interactions for all four bases. The T −3 and A −2 stacking pair are sandwiched between a tyrosine (Tyr406) and pair of histidines (His185 and His331), while T −1 is partially enclosed by tyrosine Tyr399, and G +1 stacks against phenyl­alanine Phe151 [Fig. 2 ▸(B)]. The local charge density is complementary to the bound bases, with regions of positive charge complementing O6 of G +1, and negative charge supporting N2 on the opposite side of the purine ring. Similarly, the specific methyl group C7 of T −1 is buried within a hydrophobic pocket, while the polar elements on the remainder of the pyrimidine ring, in particular O2 and O4, are complemented by corresponding polar contributions from the backbone of residues 155 and 156 [Fig. 2 ▸(E)]. Finally, there are numerous residues forming polar interactions with the functional groups of the nucleotides; serines Ser144, Ser330, Ser334 and Ser402, asparagines Asn398 and Asn186, and glutamine Gln466 [Fig. 2 ▸(B)]. It is relatively simple to conclude that the binding proclivity of the cleft, and its interaction with this region, is sequence specific. We note that this represents the only significant base interaction, involving burial within a binding site, with cognate DNA, in the structure, and that this occurs within the consensus sequence of the non-template strand. This observation is consistent with previously reported biochemical data and implies that this sequence-specific interaction determines the ΦKZ late promoter transcription start site. We note that this mode of promoter recognition is extremely different from that visualized in the AR9 nvRNAP structure, dependent on uracils in the template strand, which is mediated principally by a pocket within the AR9 σ-like subunit GP226 [Fig. 2 ▸(D), Supplementary Figure 2].

### 2.6. The modes and locations of binding to the p119L open promoter analogue suggest widely separated strand-separation and sequence-specific base recognition events

In the original paper reporting the first study on the ΦKZ nvRNAP (Yakunina *et al.*, 2015 ▸), Yakunina and colleagues investigated the selectivity of the nvRNAP for ΦKZ late promoter p119L. They demonstrated through *in vitro* experiments using purified ΦKZ nvRNAP protein samples, and purified PCR products, that the selectivity determinants for ΦKZ nvRNAP promoter recognition and transcription included both a short 4-base-pair consensus sequence identified through alignment of the known ΦKZ late promoters, and indeterminate, principally downstream, sequence requirements within the p119L promoter that did not have any significant consensus sequence or clear specificity. Our structure of the ΦKZ nvRNAP bound to a p119L promoter analogue resolves part of this dilemma. The promoter recognition and selection element determined by the 4-base-pair consensus sequence at the start site appears to be due to sequence-specific binding of this element within a pocket under the β-lobe of β-like subunit GP123. Both the location of this element at the transcription start site and the provision of consensus binding by the β-like subunit are substantial deviations from eubacterial msRNAP norms. There is no indication that the β-like subunit is capable of opening B-form DNA to access the bases for recognition. There is considerable evidence, however, that the canonical role of the σ-like subunit in promoter opening has been retained from eubacterial msRNAPs. The AR9 nvRNAP requires GP226 for both bubble opening and promoter recognition, while GP68 is required for transcription by the ΦKZ nvRNAP, and our promoter analogue structure implies roles in stabilization of upstream DNA and distortion at the bubble site consistent with strand separation. The N-terminal domain of GP68 has also been shown to be poorly ordered against the enzymatic core in the absence of DNA, becoming tightly bound in its presence (de Martín Garrido *et al.*, 2024 ▸; de Martín Garrido, Orekhova *et al.*, 2021 ▸), suggesting a role in DNA recruitment as in AR9 (Fraser *et al.*, 2022 ▸). Overall, our results imply a two-step model for open complex formation. Firstly, GP68 recruits the DNA and separates the strands. Secondly, GP123 recognizes the now open bases within the non-template strand, anchoring the nvRNAP at the start site for initiation. This implies that the two steps in promoter recognition will be spatially separated, taking place on different proteins on opposite sides of the DNA channel. They must also be temporally separated, as an open strand is required for consensus binding by GP123, given the position of the non-template strand in the DNA channel. We suggest that strand separation is carried out by GP68, supported by unknown sequence determinants, and that this is then followed by limited movement of the bubble along the DNA helix until the consensus sequence is located and recognized in a sequence specific manner by GP123. This would both explain the observation of downstream transcription determinants by Yakunina and colleagues (Yakunina *et al.*, 2015 ▸) and provide a mechanism by which the short 4 bp consensus sequence can be sufficient to specify the ΦKZ late promoter transcription start site.

### 2.7. ΦKZ nvRNAP evolution appears to have driven towards specialization, away from the eubacterial msRNAP situation towards the simpler situation typical of viral ssRNAPs

Sequence analysis and results from the ongoing proteomic expansion suggest that multi-subunit RNAPs of a similar form to the ΦKZ and AR9 nvRNAPs are widespread within jumbo-bacteriophage genomes, and that their form is generally similar to the two structurally resolved exemplars, although given their swift rate of evolution, their sequences are increasingly disparate (Fossati *et al.*, 2023 ▸). Notably, however, the equivalents of GP68 and GP123 are typically the most deviant elements of these complexes. In the proteomic study by Fossati and colleagues referred to above, for instance, no GP123 homologue could be identified. These results are in keeping with our observations for the ΦKZ nvRNAP, and suggest that conservation of the catalytic core, but variation of the promoter recognition machinery, is more widespread within the jumbo-phage family as a whole.

The identification of a full promoter specificity determination site within a β-like subunit is *extremely* unusual for a msRNAP. Almost all msRNAPs transcribe from multiple different classes of promoters under different conditions, and independent specificity subunits are required to recognize each class. The provision of specificity within the enzymatic core defeats this object, and seems likely to have been selected positively for during the relatively recent evolutionary history of the ΦKZ-like nvRNAPs. Even the AR9 nvRNAP, which is the most similar msRNAP to the ΦKZ nvRNAP that has been studied, retains the consensus binding function principally within the σ-factor-like subunit GP226. However, transcription exclusively from a single class of promoters, with only a single specificity to be accommodated, is compatible with such a shift towards specialization. Bacteriophages typically possess single-subunit RNAPs, and this is one of the reasons why these deviant bacteriophage multi-subunit RNAPs are so interesting to study. Single-subunit RNAPs are also typically characterized by transcription from a fixed promoter, often close to the initiation site, and represent a simplified transcriptional apparatus in general. The incorporation of the σ-factor-like subunit as a permanent, obligate member of the enzymatic complex (Orekhova *et al.*, 2019 ▸; de Martín Garrido, Orekhova *et al.*, 2021 ▸), and, as we can now report, the development of a sequence-specific promoter recognition pocket within the β-like subunit both act to fix the promoter that the ΦKZ nvRNAP is capable of transcribing from, resulting in a complete loss of the configurability towards different targets that is a hallmark of the msRNAPs. Together with the loss of the α and ω subunits, the key evolutionary changes that the ΦKZ nvRNAP has undergone have driven it towards increased simplicity and specificity, with greater similarity to the single-subunit RNAPs typical in other bacteriophage genomes. Without the evolutionary necessity to service multiple promoters, evolution towards similar behaviour to the single-subunit situation, even for a multi-subunit enzyme, appears to be the favoured path toward viral fitness.

## 3. Materials and methods

### 3.1. Plasmids

In order to obtain sufficient quantities of active ΦKZ nvRNAP for biochemical studies, a plasmid (pnvCo-Ex) allowing co-expression of all four ΦKZ nvRNAP β/β′-like subunits (GP55, GP74, GP71-73 and GP123) simultaneously was generated and reported previously (Orekhova *et al.*, 2019 ▸). A separate plasmid encoding GP68 was also produced in the same study (Orekhova *et al.*, 2019 ▸). Co-expression of the complete ΦKZ nvRNAP complex was accomplished by combination of both these vectors within BL21(DE3) *E. coli.* The recombinant ΦKZ nvRNAP produced using this expression system has previously been shown to be functionally equivalent to the native polymerase (Orekhova *et al.*, 2019 ▸).

### 3.2. Protein expression and purification

The full ΦKZ nvRNAP complex was expressed and purified as previously reported (Orekhova *et al.*, 2019 ▸). Briefly, BL21(DE3) *E. coli* cells were co-transformed with the two plasmids (pGP68 and pnvCo-Ex) detailed in the original study (Orekhova *et al.*, 2019 ▸). Cells were initially cultured at 37 °C to an OD_600_ of 0.7 prior to the addition of 1 m*M* IPTG to induce expression and then incubated at 22 °C for 3 h for protein production. Recombinant ΦKZ nvRNAP was then purified according to the published method (Orekhova *et al.*, 2019 ▸). A pellet containing 1 g cell mass was resuspended in 10 ml purification buffer (40 m*M* Tris–HCl pH 8.0, 10%(*v*/*v*) glycerol, 500 m*M* NaCl, 1 m*M* DTT) supplemented with 5 m*M* imidazole, and then disrupted by sonication. The resulting lysate was clarified by centrifugation at 11 000*g* (30 min, 4 °C). The supernatant was loaded onto a 1 ml HisTrap HP column (Cytiva, USA) and then washed extensively with purification buffer supplemented with 5 m*M* imidazole. Recombinant complexes were eluted through a step gradient into purification buffer supplemented with 250 m*M* imidazole. The resulting eluent was further purified through size-exclusion chromatography using a Superdex 200 Increase 10/300 GL (Cytiva, USA) in TGED buffer [20 m*M* Tris–HCl pH 8.0, 5%(*v*/*v*) glycerol, 0.5 m*M* EDTA, 1 m*M* DTT, 200 m*M* NaCl]. Fractions containing the ΦKZ nvRNAP complex were identified by Coomassie-stained SDS–PAGE, pooled and concentrated to 1 mg ml^−1^ (Amicon Ultra-4 Centrifugal Filter Unit, EMD Millipore, Merck, USA). Concentrated samples were flash frozen in liquid nitrogen as 10 µL aliquots and stored at −80 °C.

### 3.3. DNA template preparation

The p119L open promoter analogue used for cryo-EM was prepared from oligonucleotides ordered from Integrated DNA technologies (IDT). The DNA sequences were 5′-ATGAGTAATTTTAGTGAATGTATTTGCTATATTGCTATGTAGACAGTTCCCAAAAGCCTAAAGTTACAATATAGG-3′ and 5′-CCTATATTGTAACTTTAGGCTTTTGGGAACTCCTCTCATATTCCCATAGCAAATACATTCACTAAAATTACTCAT-3′ (Fig. 2 ▸; Supplementary Figure 3). For DNA template preparation, an equal volume of each oligonucleotide was mixed at a final concentration of 1 µ*M*, the mixture was then incubated at 95 °C for 5 min before cooling to 35 °C by increments of −1.0 °C min^−1^ in a thermocycler. The annealed oligonucleotides were stored at −20 °C until use. Prepared oligonucleotides were separated by native PAGE and stained with SYBR Safe DNA stain (Invitrogen, Thermo Fisher Scientific, USA) for quality control.

### 3.4. Grid preparation

Holey carbon grids (Quantifoil R 2/1 on gold 300 mesh) (Quantifoil, Germany) were washed with ultrapure water and ethyl acetate (Sigma-Aldrich, USA) to remove residual contamination from the grid production process, before extensive air drying. The washed grids were then subjected to plasma cleaning using a Basic Plasma Cleaner PDV-23 G-2 (Harrick Plasma, USA) for 15 s in air using the high mode setting. Addition of graphene oxide films was carried out according to the published protocol (de Martín Garrido, Ramlaul & Aylett, 2021 ▸) but with the replacement within the protocol of 0.03% NP-40 for 0.01% DDM.

### 3.5. Cryo-EM sample preparation

Thawed ΦKZ nvRNAP complexes were diluted to a final concentration of 0.1 mg ml^−1^ directly into 20 m*M* Tris–HCl pH 8.0, 5 m*M* MgCl_2_, 2.5 m*M* TCEP containing an equimolar concentration of the p119L open promoter analogue DNA. Complex samples were then incubated for 20 minutes at 2 °C to facilitate binding to the DNA provided. After incubation, bound ΦKZ nvRNAP complexes were adsorbed to the thin film of graphene oxide previously deposited on the surface of the grids. After application of the sample to the grids, samples were plunge-frozen in liquid ethane using a Vitrobot Mark IV (Thermo Fisher Scientific, USA) operated at 16 °C, 100% humidity, −4 blot force, 4 s waiting time and 0.5–1 s blotting time.

### 3.6. Screening of vitrification conditions

Prepared cryo-grids were clipped into autoloader clip rings (Thermo Fisher Scientific, USA) and screened on a Glacios cryo-TEM (Thermo Fisher Scientific, USA) equipped with a Falcon 4 direct electron detector and Selectris energy filter located at the Centre for Structural Biology EM facility at Imperial College London. The microscope was operated at 200 kV, 150 000-fold magnification, and over an applied defocus range of −1 to −3 µm.

### 3.7. High-resolution data collection

Selected grids were recovered from the Glacios and transferred to a Titan Krios G3i cryo-TEM (Thermo Fisher Scientific, USA) equipped with a GATAN Bio-Quantum energy filter and a K3 IS direct electron detector located at the London Consortium for cryo-EM within the Francis Crick Institute. The microscope was operated at 300 kV, 130 000-fold magnification, and over an applied defocus range of −0.75 to −2.5 µm, collecting movie images with a total exposure of 50 e^−^ Å^−2^. A combined total of 20 670 movies were recorded using *EPU* (Thermo Fisher Scientific, USA) with an object pixel size of 0.66 Å pixel^−1^ (Supplementary Figure 4; Supplementary Table 1).

### 3.8. Image processing

Movie frames were aligned and dose-weighted using *MotionCor2* (Zheng *et al.*, 2017 ▸), while CTF parameters were estimated with *CTFFIND4* (Rohou & Grigorieff, 2015 ▸). Micrographs displaying irregular Thon rings, excessive ice or insufficient high-resolution information according to power spectrum Thon-ring fitting were discarded, leaving 20 215 micrographs for further processing (Supplementary Figure 4). Initially, 4 178 816 sites were selected semi-automatically using *BATCHBOXER* (Tang *et al.*, 2007 ▸), and particle images were extracted at 3.96 Å pixel^−1^ before classification into 2D averages with *RELION 4* (Kimanius *et al.*, 2021 ▸) leaving the CTF uncorrected up to the first peak. Particles leading to high-resolution class averages (1 793 053) were retained for further refinement (Supplementary Figure 5). This set of particles was further classified into four 3D classes. One of the classes containing 512 009 particles had clearly defined density for bound DNA and peripheral regions of the complex (Supplementary Figure 5). These particles were re-extracted at 0.99 Å pixel^−1^ for gold-standard refinement in *RELION*. CTF and aberration refinement (including beam tilt, anisotropic magnification and per-particle CTF estimation) were also performed on this set of particles using *RELION 4* (Kimanius *et al.*, 2021 ▸), leading to a 2.50 Å reconstruction according to an independent half-set FSC of 0.143 (Supplementary Figure 6). Particles contributing to this high-resolution reconstruction were further sorted to improve the density of the peripheral DNA and GP68 NTD regions. 3D classification without an angular search (--skip_align) separated two major classes differentiated by the position of the trigger-loop insertion domain, one of which, comprising 329 400 particles, demonstrated stronger density for the DNA and peripheral regions (Supplementary Figure 6). Further gold-standard refinement of this set of particles yielded a final high-resolution reconstruction reaching 2.59 Å resolution according to an independent half-set FSC of 0.143 (Supplementary Figures 6 and 7).

### 3.9. Modelling and coordinate refinement

The previously published ΦKZ nvRNAP transcription complex structure (8que) (de Martín Garrido *et al.*, 2024 ▸) supplemented with a new model for the N-terminus of GP68 (1–318) generated using *AlphaFold3* (Abramson *et al.*, 2024 ▸) was employed as the initial model for rebuilding. The molecular model was then rebuilt with *COOT* (Emsley & Cowtan, 2004 ▸; Emsley *et al.*, 2010 ▸). The density for the majority of the complex (GP55, GP71-73, GP74, GP123-NTD and CTD, and GP68-CTD) is well resolved. Side-chain conformers can clearly be assigned in the core, and in places the holes within aromatic rings can be seen, while the backbone within the periphery can be readily traced even if side chains are disordered (Supplementary Figure 9). As in our previous structure (8que) the intermediate domain of GP123 exhibits rotational smearing against the core of the complex, preventing the peripheries of this region from being reliably built. In this structure, however, the NTD of GP68, which is similarly weakly bound, is better resolved than was previously the case. The density for the majority of GP68 residues 1–150 is poorly resolved; however, better density can be observed for residues 151–318, which allowed model building and refinement (Fig. 1 ▸). The DNA was initially traced through the entire reconstruction at low resolution using a filtered map (Supplementary Figure 8) to ensure that the offsets and lengths of poorly ordered regions matched those expected. The consensus motif, bound between the GP123 intermediate and NT/CT domains, was sufficiently well resolved to allow it to be assigned directly based on the density of the bases (Supplementary Figure 9). The region of the template strand ordered along the side of the DNA channel within the core of the complex was also sufficiently well resolved to allow assignment, and the register of the more peripheral B-form double-helical DNA outside of the DNA channel was assigned based upon this region, to which it was connected by resolved phosphate backbone, and base pairing for the other strand. The atomic model was refined against the gold-standard output map using *phenix.real_space_refine* (Afonine *et al.*, 2012 ▸), with the application of secondary-structure restraints for the intermediate domain of GP123, the NTD of GP68 and the ordered DNA. The resulting model had respectable quality metrics, most notably a 99th percentile *MolProbity* score (Supplementary Table 1).

## 4. Related literature

The following references are cited in the supporting information: Cardone *et al.* (2013 ▸), Tan *et al.* (2017 ▸).

## Supplementary Material

Supplementary figures and table. DOI: 10.1107/S2052252525009273/rq5015sup1.pdf

PDB reference: PhiKZ non-virion RNA polymerase bound to a p119L open promoter analogue, 9rjs

EMDB reference: PhiKZ non-virion RNA polymerase bound to a p119L open promoter analogue, EMD-54012

## Data Availability

The cryo-EM density map resolved for the ΦKZ nvRNAP in complex with the p119L open promoter analogue has been deposited in the Electron Microscopy Data Bank under accession code EMD-54012, while the corresponding molecular model has been deposited in the Protein Data Bank as PDB ID 9rjs.
