# Supplementary material for: Structure of the bacteriophage PhiKZ non-virion RNA polymerase bound to a p119L open promoter analogue
Source: IUCrJ. 2026 Jan 1;13(Pt 1):31–43. doi: 10.1107/S2052252525009273 (PMC12809450; doi:10.1107/S2052252525009273)
Supplement: Supplementary file 1 [file m-13-00031-sup1.pdf]

IUCrJ

Volume 13 (2026)

Supporting information for article:

Structure of the bacteriophage PhiKZ non-virion RNA polymerase bound to a p119L open promoter analogue

Chao-Sheng Chen, Natàlia de Martín Garrido, Maria Yakunina and Christopher H S Aylett

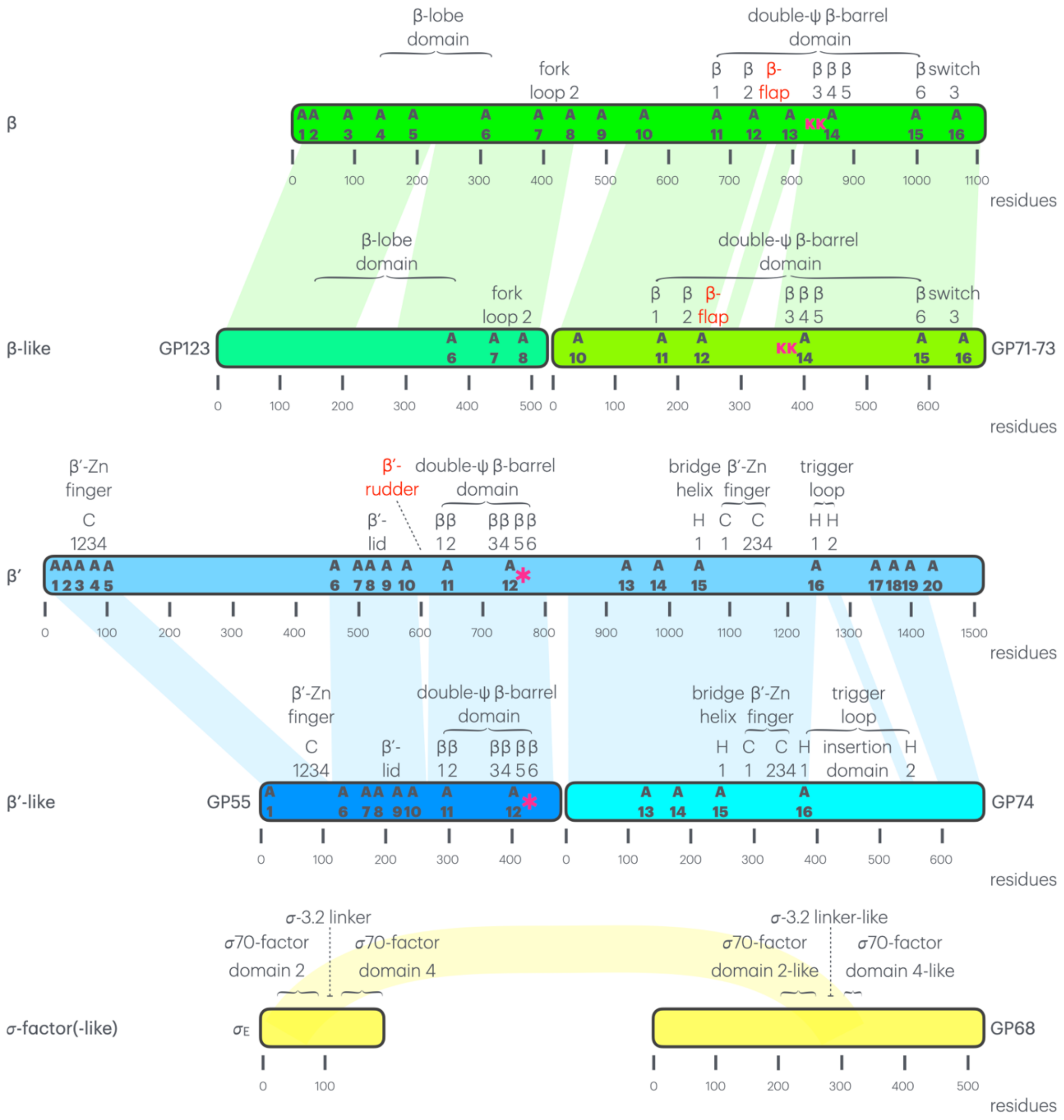

Supplementary Figure 1: Schematic showing conservation between bacteriophage ΦKZ nvRNAP subunits and those of eubacterial msRNAPs. The primary structures of the ΦKZ nvRNAP subunits and eubacterial (*T. thermophilus* β and β' and *E. coli* σ_E) msRNAP subunits are shown with regions of structural conservation connected by transparent blocks. Key features of the msRNAP enzymes are labelled appropriately. The α and ω subunits are not shown as the ΦKZ nvRNAP does not possess homologues. Conserved regions originally detected by sequence similarity are labelled (β: A1-16 / β':A1-20) according to the Lane and Darst nomenclature, as assigned in Yakunina, *et al.* 2015. Further assignments have been made according to atomic structure. Short insertions or rearrangements below 30 residues have been excluded to avoid fragmentation, except where functionally relevant: e.g. the lack of the flap-tip helix within the β-like subunit GP71-73. Red text has been used to highlight notable losses or differences from the canonical msRNAP structure, the active site has been highlighted by an asterisk, and lysine residues contributed by the β(-like) subunit are indicated by K's.

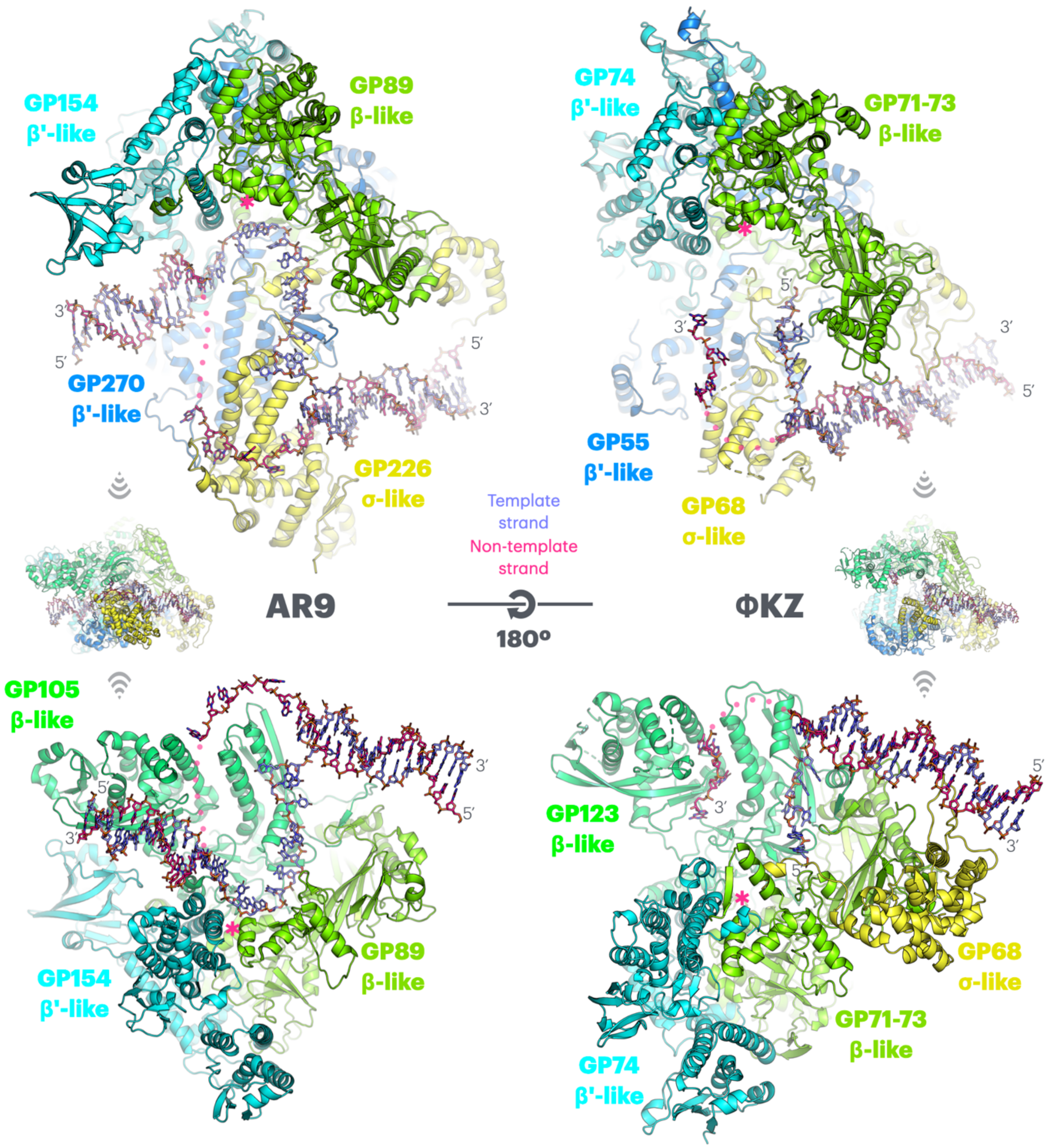

Supplementary Figure 2: Comparison of the DNA channels of the ΦKZ and AR9 nvRNAPs. The structures of the ΦKZ nvRNAP (right) and AR9 nvRNAP (left) are shown from either side of the nucleic-acid binding cleft with the oligonucleotides in place, but obscuring regions removed, to aid comparison of the two non-canonical msRNAPs resolved to date. The full structure is shown inset in each panel, to indicate the direction of the view provided in each case with an expanding cone. Colours are as in Figure 1 of the manuscript, the active site location is also indicated with a magenta asterisk.

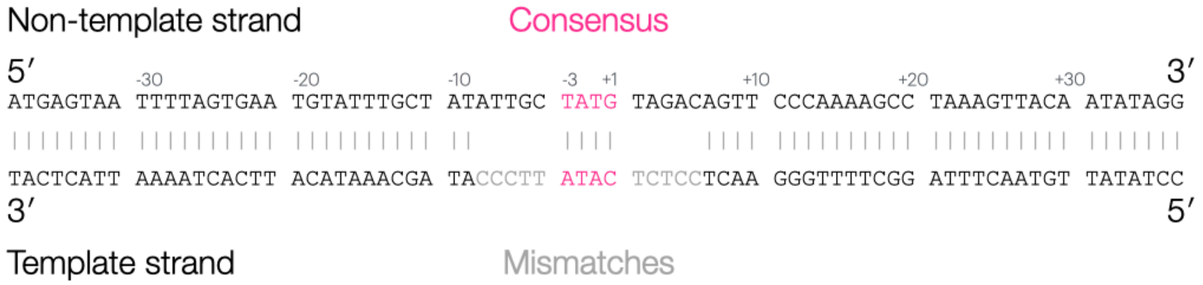

Supplementary Figure 3: The p119L double-bubble open promoter analogue DNA template. (A) The form and sequence of the p119L double-bubble open promoter analogue DNA template. The short consensus region at the start site is shown in magenta, while the mismatches incorporated into the template strand to create bubbles and generate a stable open promoter analogue are shown in grey.

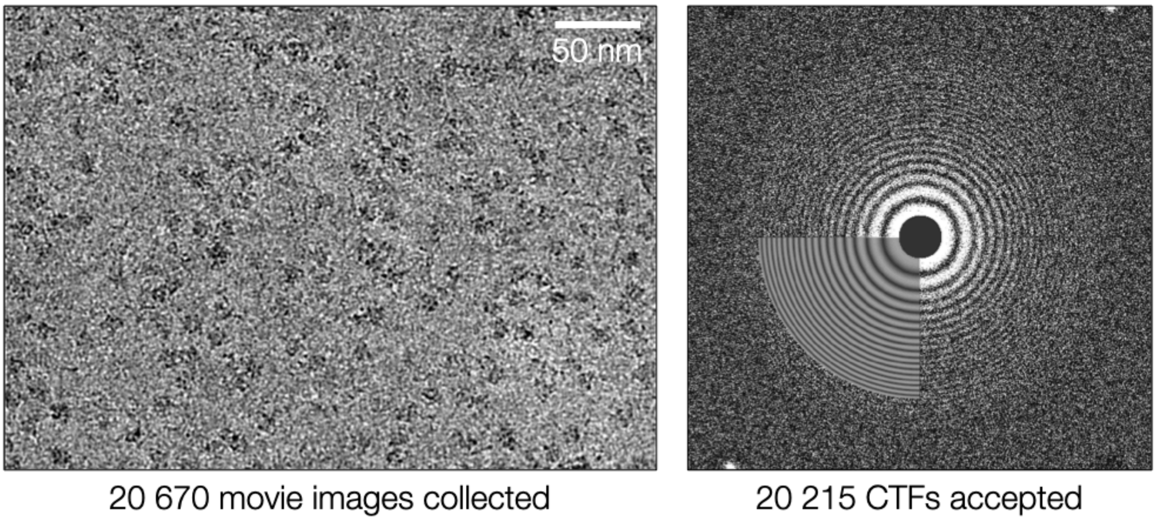

Supplementary Figure 4: High-resolution data collection for ΦKZ nvRNAP complexes bound to the p119L open promoter analogue. (Left) Representative micrograph of nvRNAP complexes bound to the p119L open promoter analogue adsorbed to a graphene oxide film deposited on a gold grid. (Right) CTFFIND (Rohou & Grigorieff, 2015) comparison between Thon rings simulated from the estimated CTF parameters (bottom left quadrant) and an experimental power spectrum from a micrograph with a ~2.5 Å resolution limit.

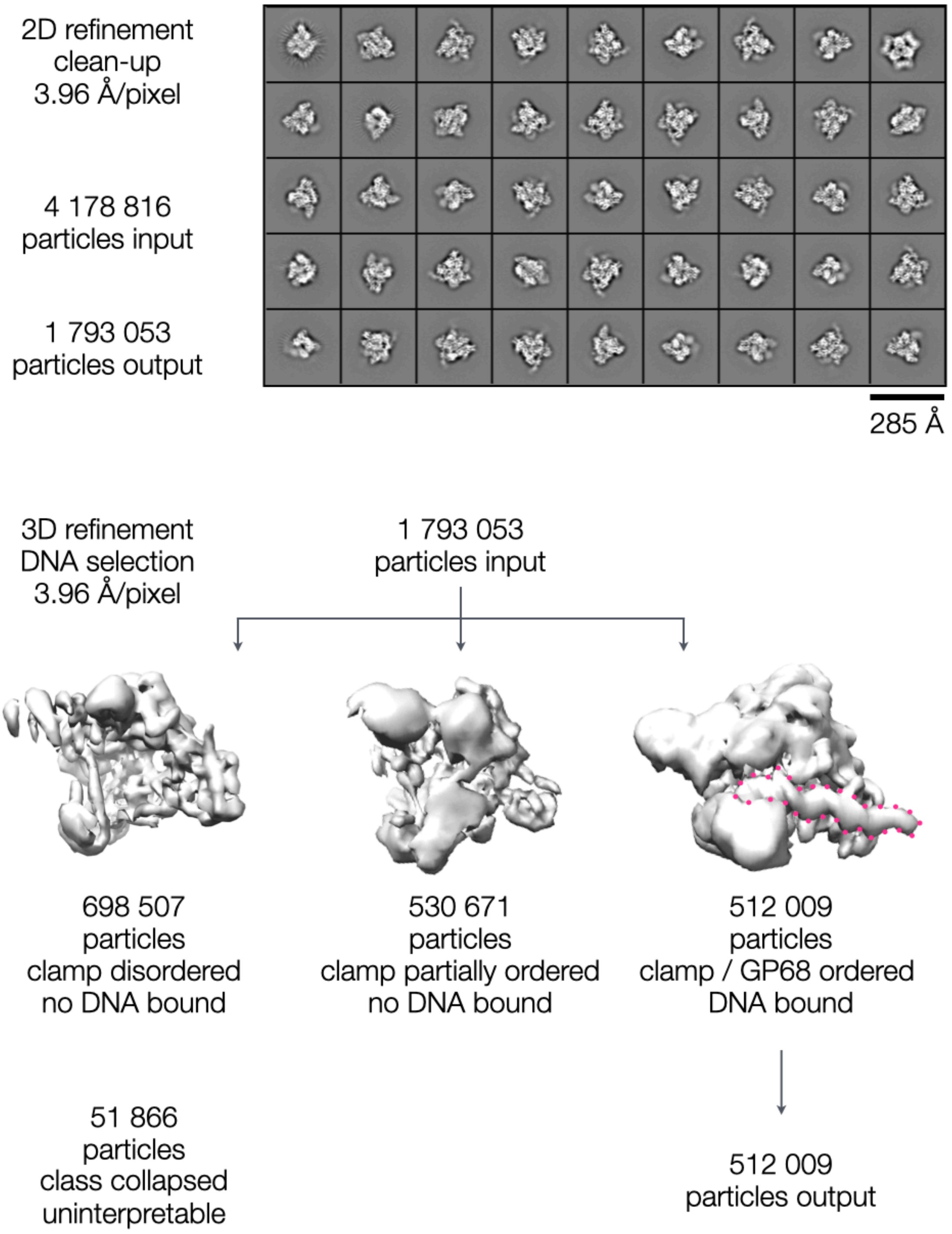

Supplementary Figure 5: Initial data processing scheme for the ΦKZ nvRNAP complex bound to the p119L open promoter analogue at 3.96 Å/pixel. Flow-chart depicting the data processing procedure, with images of the 2D and 3D classes recovered at each stage of the procedure. Particle numbers retained at each stage of the process are indicated either adjacent to, or below each reconstruction. The bound DNA visualised in the final selected class is highlighted with a magenta dotted outline.

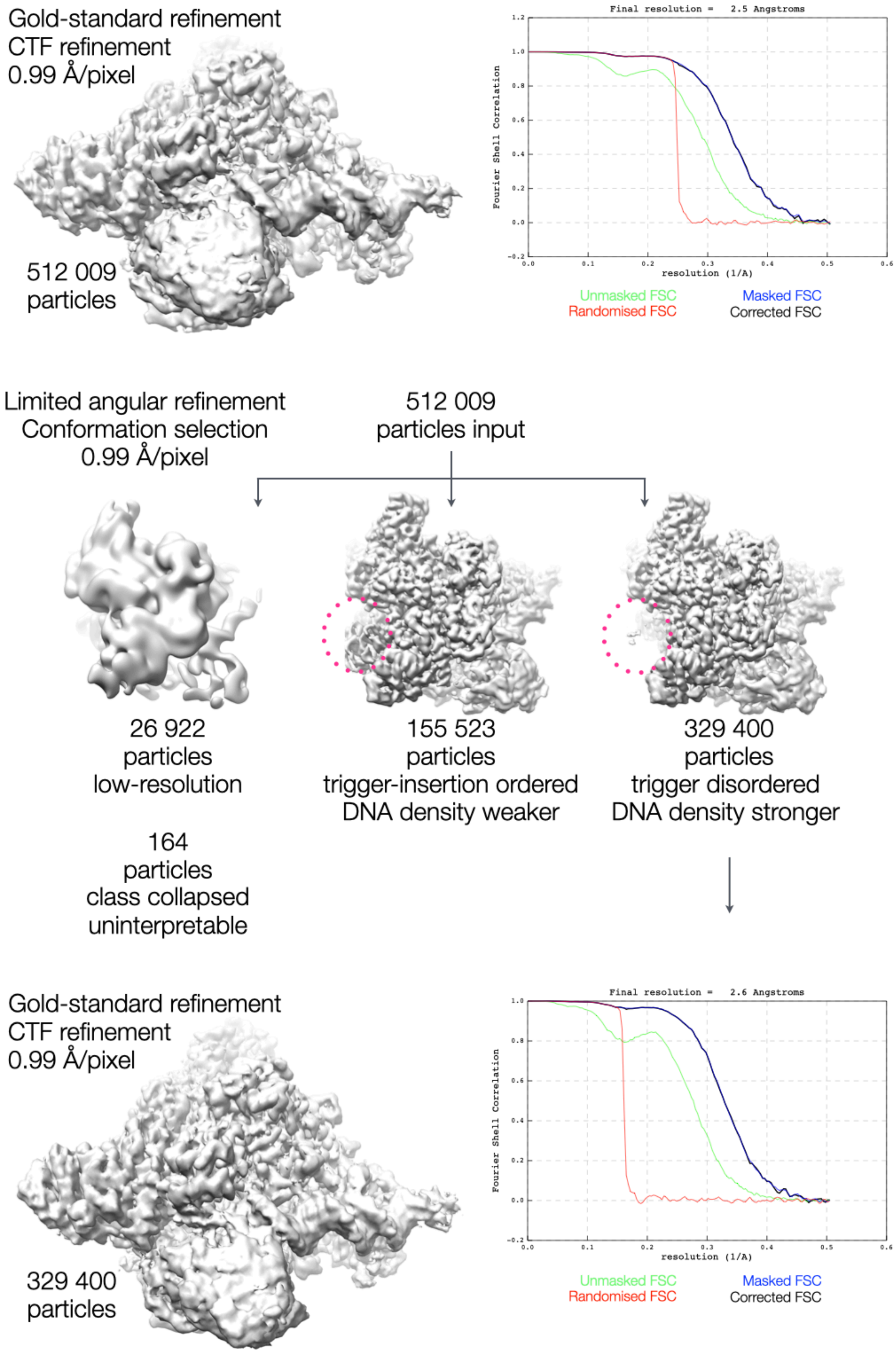
Gold-standard refinement
CTF refinement
0.99 Å/pixel
512 009
particles
Final resolution = 2.5 Angstroms
Fourier Shell Correlation
resolution (1/A)
Unmasked FSC
Masked FSC
Randomised FSC
Corrected FSC
Limited angular refinement
Conformation selection
0.99 Å/pixel
512 009
particles input
26 922
particles
low-resolution
155 523
particles
trigger-insertion ordered
DNA density weaker
329 400
particles
trigger disordered
DNA density stronger
164
particles
class collapsed
uninterpretable
Gold-standard refinement
CTF refinement
0.99 Å/pixel
329 400
particles
Final resolution = 2.6 Angstroms
Fourier Shell Correlation
resolution (1/A)
Unmasked FSC
Masked FSC
Randomised FSC
Corrected FSC

Supplementary Figure 6: Final data processing scheme for the ΦKZ nvRNAP complex bound to the p119L open promoter analogue at 0.99 Å/pixel. Flow-chart depicting the data processing procedure, with images of 3D reconstructions recovered at each stage of the procedure. The RELION postprocessing FSC curves output on resolution determination by the package (Kimanius *et al.*, 2021) are shown adjacent each gold-standard reconstruction. Particle numbers retained at each stage of the process are indicated either adjacent or below each reconstruction. The trigger insertion domain identified in multiple conformations is highlighted with an outline of magenta dots. Both the 2.50 Å and 2.59 Å reconstructions were used during interpretation of the density; the latter is deposited in the EMDB.

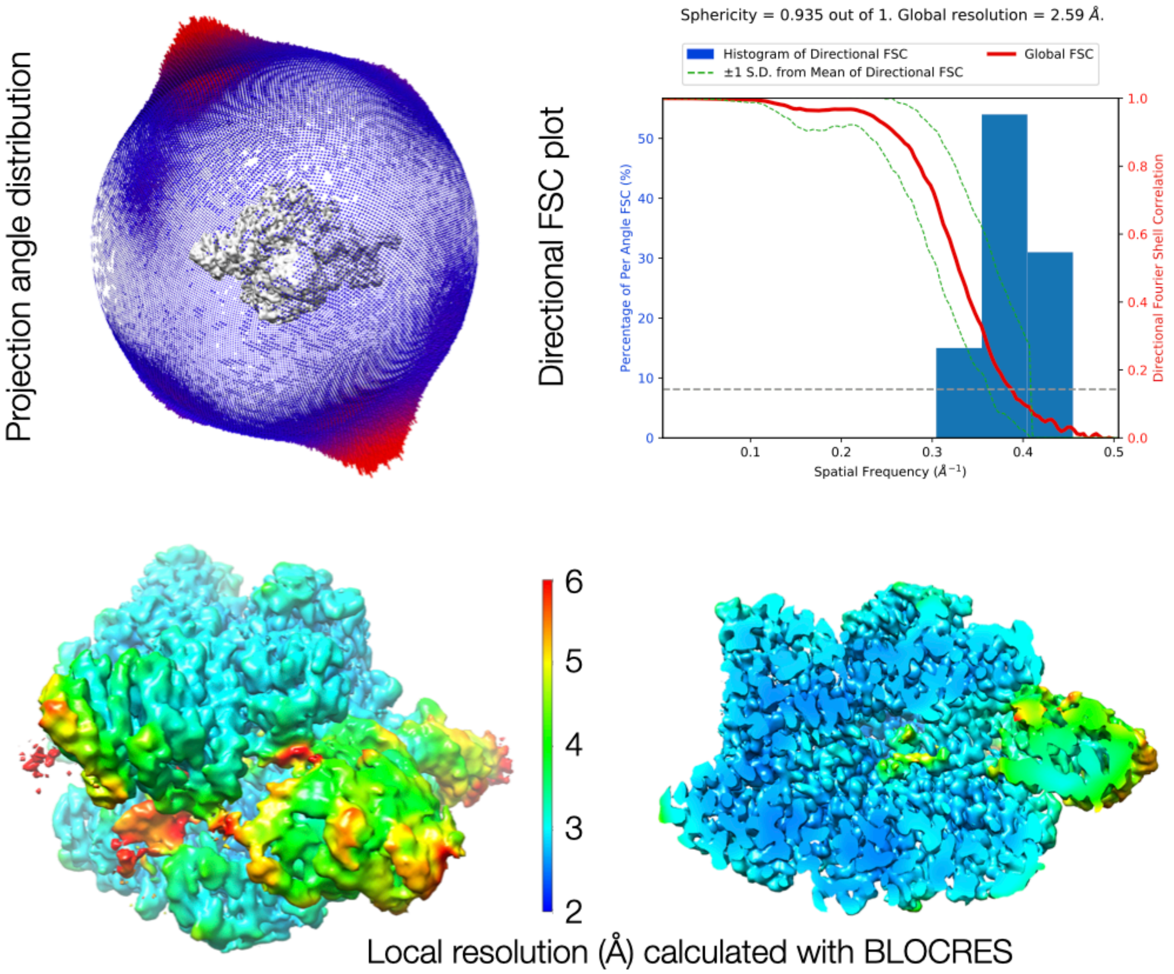

Supplementary Figure 7: Anisotropy and local resolution of the final reconstruction of the ΦKZ nvRNAP complex bound to the p119L open promoter analogue. (Top left panel) Angular distribution of projections during the final iteration of the 2.59 Å reconstruction. (Top right panel) Directional FSC curve for the 2.59 Å reconstruction calculated using the 3D FSC server (Tan *et al.*, 2017), showing little resolution anisotropy. (Bottom) Local resolution map of the 2.59 Å reconstruction calculated using BLOCRES (Cardone *et al.*, 2013). The bottom left panel shows the reduced peripheral resolution visible on the conformationally variable side of the molecule bearing the DNA and the N-terminus of GP68. The bottom right panel shows a slice through the core of the molecule revealing well-ordered ~2.5 Å resolution density.

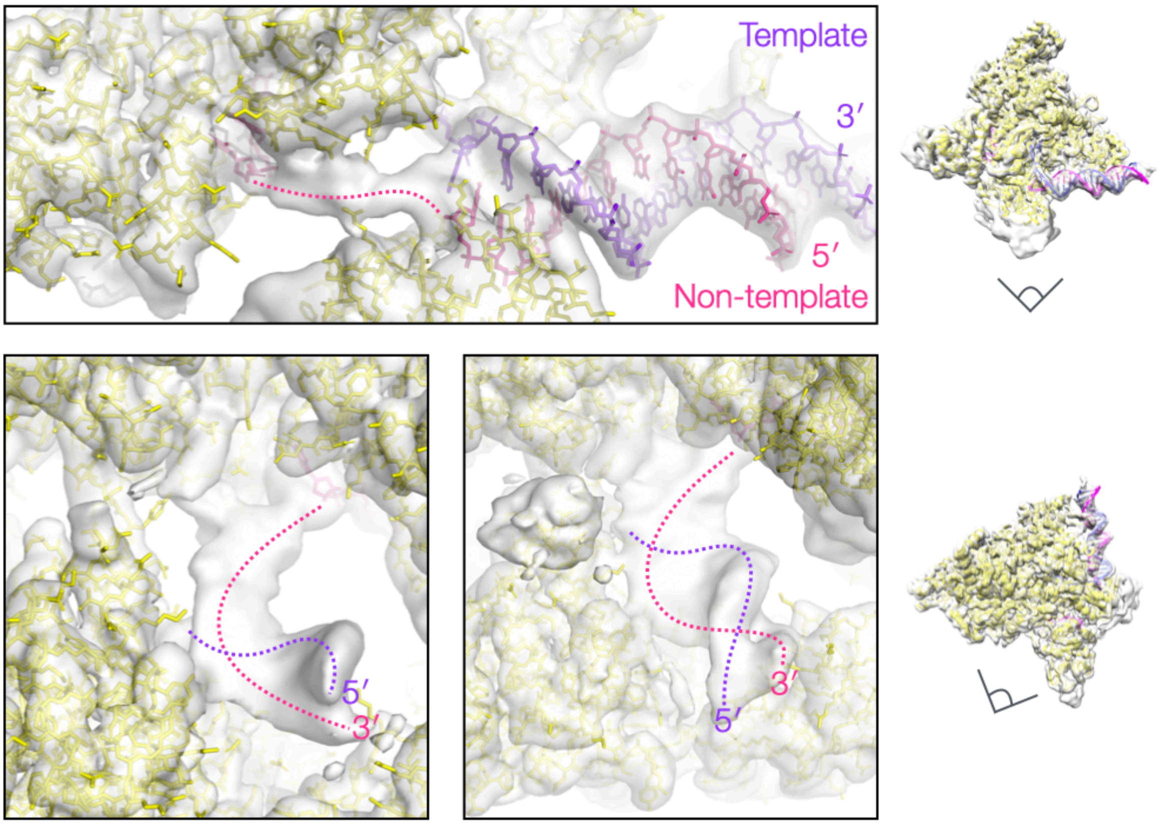

Supplementary Figure 8: Gaussian filtration (sigma of 1 Å) of the 2.59 Å density allows the path of the DNA to be traced through the active site beyond the regions which can be built directly. Upper panel: the path of the non-template strand can clearly be traced from the B-form region to the consensus sequence bound by GP123. Lower panels: the path of the non-template strand can be clearly traced leaving GP123 and joining the template strand to form a B-form helix occupying the DNA entrance channel in the same fashion as resolved in our previous structure PDB ID 8QUE and similar to that visualised in AR9 RNAP PDB ID 7S01 (de Martin Garrido *et al.*, 2024, Fraser *et al.*, 2022). Overviews of the entire structure are shown on the right with the viewing point indicated to aid orientation.

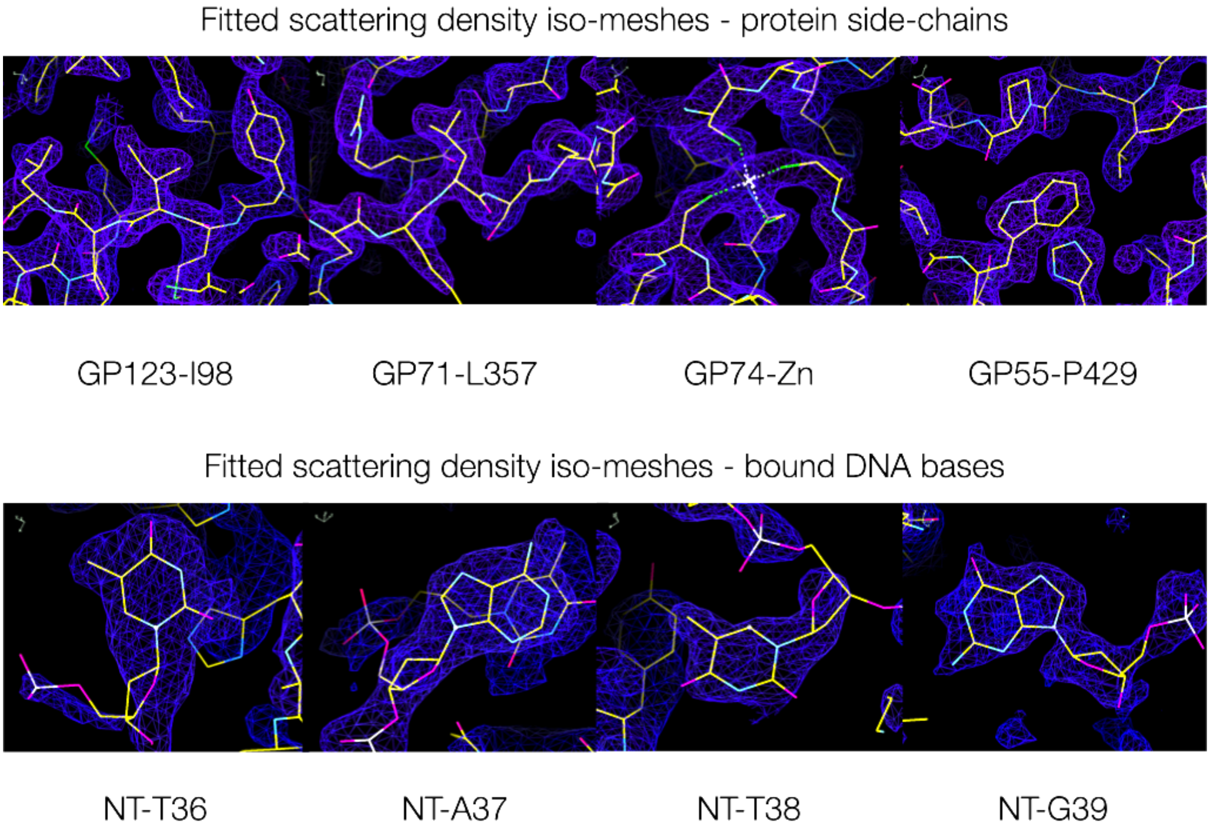

Supplementary Figure 9: Sample density for the ΦKZ nvRNAP complex bound to the p119L open promoter analogue. Upper panels: examples of side-chain density from the well-ordered core regions of the 2.50 Å structure. The density is high-quality, side chains can be unambiguously interpreted, and holes within rings are visible in some regions (e.g. right panel). Lower panels: comparatively lower-local resolution density of the key nucleotide bases within the consensus sequence, showing that the base densities are interpretable at a level beyond that simply allowing the model builder to distinguish between purines and pyrimidines so that the consensus sequence and register can be assigned.

Magnification (×)	130 000
Voltage (kV)	300
Electron exposure ($e^{-}/Å^2$)	50
Defocus range (µm)	-0.75 to -2.50
Pixel size (Å/pix)	0.66
Symmetry imposed	C1
Initial number of particles	4 178 816
Final number of particles	329 400
Map resolution (Å) at FSC = 0.143	2.59
Model-Map CC Mask / Model-Map CC Volume / Mean B-factor	0.90 / 0.90 / 104.82
Molprobity overall score / clash score	1.16 / 2.91
RMS deviations in bond lengths (Å) / angles (°)	0.003 / 0.410
Ramachandran favoured / allowed / disallowed (%)	97.6 / 2.4 / 0.0
Rotamers favoured / allowed / disallowed (%)	95.1 / 4.8 / 0.1

Supplementary Table 1: Collection parameters and model refinement statistics for the cryo-EM structure of the ΦKZ nvRNAP bound to the p119L DNA open promoter analogue. Data were acquired on a Titan Krios G3i cryo-TEM (Thermo Fisher Scientific, USA) equipped with a GATAN Bio-Quantum energy filter and a K3 IS direct electron detector located at the London Consortium for Cryo-EM within the Francis Crick Institute.

References

Cardone, G., Heymann, J. B. & Steven, A. C. (2013). *J Struct Biol* **184**, 226-236.

de Martin Garrido, N., Chen, C. S., Ramlaul, K., Aylett, C. H. S. & Yakunina, M. (2024). *J Mol Biol* **436**, 168713.

Fraser, A., Sokolova, M. L., Drobysheva, A. V., Gordeeva, J. V., Borukhov, S., Jumper, J., Severinov, K. V. & Leiman, P. G. (2022). *Nat Commun* **13**, 3526.

Kimanius, D., Dong, L., Sharov, G., Nakane, T. & Scheres, S. H. W. (2021). *Biochem J* **478**, 4169-4185.

Rohou, A. & Grigorieff, N. (2015). *J Struct Biol* **192**, 216-221.

Tan, Y. Z., Baldwin, P. R., Davis, J. H., Williamson, J. R., Potter, C. S., Carragher, B. & Lyumkis, D. (2017). *Nat Methods* **14**, 793-796.

Yakunina, M., Artamonova, T., Borukhov, S., Makarova, K. S., Severinov, K. & Minakhin, L. (2015). *Nucleic Acids Res* **43**, 10411-10420.
